# Dietary Intake of Toxic Heavy Metals with Major Groups of Food Products—Results of Analytical Determinations

**DOI:** 10.3390/nu14081626

**Published:** 2022-04-13

**Authors:** Wojciech Koch, Marcin Czop, Katarzyna Iłowiecka, Agnieszka Nawrocka, Dariusz Wiącek

**Affiliations:** 1 Department of Food and Nutrition, Medical University of Lublin, 4a Chodźki Str., 20-093 Lublin, Poland; katarzyna.ilowiecka@umlub.pl; 2 Department of Clinical Genetics, Medical University of Lublin, 11 Radziwiłłowska Str., 20-080 Lublin, Poland; marcin.czop@umlub.pl; 3 Institute of Agrophysics, Polish Academy of Sciences, 4 Doświadczalna Str., 20-290 Lublin, Poland; a.nawrocka@ipan.lublin.pl (A.N.); d.wiacek@ipan.lublin.pl (D.W.)

**Keywords:** food, dietary intake, heavy metals, ICP-OES

## Abstract

Food contains a complex matrix of various substances, including essential nutrients, non-nutritive substances, and toxins, including metals. The main purpose of the study was to evaluate the contribution of major groups of food products to an overall intake of toxic heavy metals (Cd, Pb, Hg, and Ni) using a combination of the 24-dietary recall technique, the ICP-OES (Inductively Coupled Plasma Optical Emission Spectrometry) method, and chemometric tools. The obtained results reveal that there is a high potential risk of developing nephrotoxicity through the dietary intake of Pb in the case of both genders. The dietary intake determined for other elements (Cd, Hg, and Ni) was far below the limits established by European Food Safety Authority (EFSA). Principal Component Analysis (PCA) supported analytical determinations and revealed that cereals and vegetables were major contributors to a total intake of Cd (39.6 and 17.4% of the total exposure, respectively), Ni (40.4 and 19.3%), and Hg (16.8 and 19.6%), while water and beverages were major dietary sources of Pb (31% of the total daily intake). In contrast, eggs, fats and oils, and milk and dairy products provided the smallest amounts of Cd, Pb, and Ni. Despite containing high amounts of Hg, considering very low consumption, fish were not found to be an important source of this element.

## 1. Introduction

Food is a major source of nutrients for the body, but it also contains other components, such as non-nutritive substances, which are not only unnecessary but may be harmful to the organism [1]. Among many various minerals, which are consumed in a daily diet, special concern should be paid to toxic heavy metals, such as Cd, Pb, Hg, or Ni. These elements do not possess any known positive function, being even toxic at relatively low concentrations. As, a metalloid present in food, is considered a typical toxic contaminant present in food products, and, therefore, its content is regulated by appropriate EU legislation [2,3]. For people who are not professionally exposed to these elements, food is the main source of exposure. Despite the fact that these elements are present in food products in relatively low concentrations, according to the current knowledge, they pose a serious threat, especially taking into account chronic exposure [2,3]. The European Commission (EC), in the relevant regulations, established the acceptable limits for the content of heavy metals in food products [4,5]. Therefore, there is a constant need to monitor their concentration in various foods, as well as their dietary intake with daily food rations. Indeed, studies of such a type are conducted, and every year the world’s scientific literature is enlarged by new publications on this subject [6,7,8,9]. However, food products, dietary habits, and food patterns are evolving. New products appear on the market every day, especially functional foods, the global market of which has been predicted to be worth $192 billion. This group of products, often indicated as ‘natural health products’ or ‘healthy foods’, contains mainly foods that were fortified with bioactive constituents or the chemical composition of which was changed to modify or increase their nutritional/biological value. This causes changes in the content of individual components in the product, also in relation to minerals, including toxic heavy metals, compared to the initial product [10,11]. Moreover, eating habits are still changing, and the current diet of Western, industrialized societies is significantly different, not only compared to ancient or medieval times but also to the status two or three decades ago [12]. Another issue is not only the quality or amount of food consumed but the way it is consumed. This also evolves, and people today consume their meals faster, without proper chewing, which results in consuming more food and significantly contributes to the development of overweight and obesity [13]. It also directly influences the intake of various elements, including heavy metals [14]. Therefore, studies focusing on the evaluation of food consumed and analyzing the level of intake of different food components are crucial to evaluate dietary habits, new trends, and social interventions, and in the case of toxic metals, also to evaluate the risk associated with dietary intake and exposure to these elements. The World Health Organization (WHO) encourages countries to undertake a national dietary survey (NDS) to track and evaluate the intake of macro- and micro-elements [15]. However, an appropriate evaluation of the dietary intake of different nutrients and non-nutrients is hard to conduct, as the food matrix is very complex, and many components are present at the level of ppm or even ppb (trace and toxic elements). Another issue is the assessment of elemental intake. The majority of studies have been based on calculations using specific databases regarding the content of particular nutrients in food products. Although such studies are quite cheap and easy to perform, the obtained results are often far from displaying a real intake, because many products present on the market contain different levels of nutrients in comparison to the levels stated in a database. This is due to many factors. First of all, food products are constantly changed, and food composition databases may not accurately reflect fortification (with iron or iodine) [16]. Secondly, some databases are not updated on time, which means that they do not keep up with changes related to product reformulation. Hutchinson et al. revealed that the values regarding the content of trans fatty acids in the database may be higher than those found in products available on the market [17]. In the case of toxic heavy metals, another problem is the availability of a special database on their content in food. In some countries, such an up-to-date database exists (e.g., Denmark), and the computational assessment of heavy metals intake with food, despite its numerous disadvantages, is feasible [9]. However, in many countries, such databases do not exist or are out of date. In Poland, the existing database was based on the determinations from the 1980’s and, therefore, it is well out of date [2]. Thus, in Poland, the only way to evaluate the dietary intake of various toxic heavy metals is through direct chemical analysis. There have been several such studies performed in Poland in recent years, but a thorough analysis, including the participation of major food products in the total dietary intake of toxic heavy metals, was last conducted over 20 years ago by Marzec and Schlegel-Zawadzka [18]. Therefore, there is a strong need to evaluate the actual dietary exposure to heavy metals among the adult population of Poland and to indicate the main contributors to the overall daily intake. Studies based on the analytical determinations of elements in daily food rations, although difficult to conduct and expensive, more properly reflect the actual dietary intake and current exposure to toxic metals, in comparison to data based on calculations [6,7,8,19].

The main objective of this study was to evaluate the dietary intake of four toxic heavy metals (Cd, Pb, Hg, and Ni) in the main food groups among young Polish adults. The first hypothesis stated that cereals would be a major or main food group, contributing in the highest degree to the overall intake of investigated metals. The second hypothesis assumed that, since the Lublin region in which the study was conducted is an agricultural one, without heavy industry, the daily intake of all toxic elements would be below the safe levels of intake (PTWI or TDI). Selected chemometric tools (ANOVA and PCA) were used to process the obtained data, to indicate the main contributors, which significantly and to the highest extent participate in the total intake of particular elements, and to reveal important correlations between metals and food consumed. A comparison between the obtained results and worldwide trends regarding the dietary intake of Cd, Pb, Hg, and Ni, and their concentration in various foods, was an additional goal of the study. 

The study combined various methods to obtain reliable data, which were developed and validated before [8,20]. Briefly, data regarding food intake were assessed using the 24-dietary recall technique, which is considered the most appropriate self-report questionnaire for nutritional data gathered no earlier than during the past 3 days [16,21,22,23,24]. The obtained results were processed using Dieta 5.0 Software (National Institute of Public Health, Warsaw, Poland), which is the official software used to assess the quality of nutrition in Poland, as recommended by NIPH. The processed data were used in the reconstruction of particular food groups (for both genders), in which toxic heavy metals were determined using the ICP-OES method. 

## 2. Materials and Methods

### 2.1. Reagents (Chemicals)

All reagents were of Suprapur Grade and were bought from Merck (Darmstadt, Germany). High purity deionized water (resistivity of 18.2 MWcm) obtained using an Ultrapure Millipore Direct-Q-R 3UV (Millipore, Bedford, MA, USA) was used throughout the analysis. Quartz crucibles and polypropylene recipients were applied for the digestion and digest storage, respectively. 

### 2.2. Sampling 

The presented results were part of a bigger study conducted to evaluate nutrition, dietary habits, and dietary intake of various components among young adults from the Lublin region, using nutritional databases, analytical methods, and their combinations. The study used various nutritional tools to assess the dietary intake of toxic heavy metals with food as accurately as possible. For this reason, the 24-dietary recall technique, food databases, and analytical determinations of toxic heavy metals using ICP-OES were used.

A total of 579 healthy participants (274 women and 305 men) were involved in the study. All the participants were volunteers and the investigations conducted were totally anonymous. A detailed description of the studied population was previously presented [20]. The study was performed between October 2016 and March 2018. The participants were asked to fulfill previously validated questionnaires containing qualitative and quantitative parameters of their diets. Only midweek days were taken into consideration. All the respondents were informed about the purpose of the study, gave their oral consent, and were assured of the anonymity of the study. The participants were interviewed in the Department of Food and Nutrition of the Medical University in Lublin. The collected data were checked and only corrected questionnaires were included in the study. The obtained data were processed using two nutritional computer programs—Dieta 5.0 Software (National Institute of Public Health, Warsaw, Poland) and Dietetyk 2006 (Jumar, Poznań, Poland). These were used in order to average the data and assign products to particular main groups, separately for women and men. According to Polish standards recommended by the NIPH, all food products were divided into the major 12 food groups (Table 1).

In the next step, data obtained from computer analysis were used to reconstruct all average food groups, separately for women and men. Duplicates were prepared using food products from the retail market of the Lublin region and its province, i.e., the home region for people participating in the study. For each product, different brands have been mixed to compose a representative sample. For each food group, three separate duplicates were prepared. In total, 216 food samples were subjected to analytical determinations (2 genders × 12 food groups × 3 reconstructions × 3 repetitions).

All food items were prepared in the laboratory according to the local cooking practices which were previously described in detail [8,20].

### 2.3. Analytical Determination of Cd, Pb, Hg and Ni Using ICP-OES

Homogenized food samples were digested according to previously described protocols [8,20]. Concentrations of Cd, Pb, Hg, and Ni were determined using the ICP-OES method with an iCAP 6500 Duo, an inductively coupled plasma optical emission spectrometer (Thermo Fisher Scientific, Waltham, MA, USA), which was controlled by PC-based iTEVA software. The following instrumental settings were used: an RF generator power of 1150 W, an RF generator frequency of 27.12 MHz, a coolant gas flow rate of 16 L·min^−1^, a carrier gas flow rate of 0.65 L·min^−1^, an auxiliary gas flow rate of 0.4 L·min^−1^, maximum integration time of 15 s, a pump rate of 50 rpm, and axial viewing configuration flush time of 20 s. All determinations were made in triplicates. Calibrations were performed using multi-element standards CCS-4 and CCS-6 (100 g/mL in 7% HNO3; Inorganic Ventures, Christiansburg, VA, USA).

The method was previously validated [8,20]; however, it was once again checked for its accuracy and precision in the determination of heavy metals in food samples. For this purpose, a mixture of flour and milk powder (70:30 m/m), which was fortified with known concentrations of various elements, was used as reference material and subjected to the same analytical protocol as the studied samples. The analysis of the reference material was performed in six replicates and the obtained results were presented in Table 2.

### 2.4. Statistical Analysis

Statistical analysis was performed using Statistica 13.3 (StatSoft, Kraków, Poland) and Graph Pad Prism 7 (Graph Pad Software, San Diego, CA, USA). Data were analyzed for normal distribution using the Shapiro–Wilk test. Statistically significant differences in heavy metals’ content between particular food groups and genders were analyzed using two-way ANOVA (with two qualitative factors) followed by Tukey’s test. To reduce the number of variables and to detect the structure of relationships between them, PCA with Varimax rotation was used. All results are presented as mean ± standard deviation (SD). The level of statistical significance was set at *p* ≤ 0.05.

## 3. Results and Discussion

The applied methodology allowed for accurately estimating the dietary intake of the investigated elements, reflecting the actual food conditions in the local area, considering the participation of particular groups of the main food products in the total daily intake. The validity of combining the methods described above as appropriate to assess the precise intake of trace elements has already been proven [2,8,20], which allows us to assume that the obtained results are very close to the actual dietary exposure to Cd, Pb, Hg, and Ni among young adults in Poland. Moreover, the presented results shed new light on the distribution of intake in the context of actual dietary habits of young Poles regarding the exposure to toxic heavy metals.

As was previously mentioned [20], solid foods, vegetables, cereals, meat, and meat products, as well as dairy products, were consumed in the highest amounts among both genders. In contrast, very low consumption of fish, among both women and men, should be noticed. Among liquid foods, black tea infusions were by far consumed in the highest amounts, followed by soups, coffee, and fruit juices. Black tea is very popular in European countries, and it was previously described that infusions prepared from this plant are the most popular beverage in the world, including Poland, after water [25,26]. Since black tea infusions may be a rich source of some elements (e.g., Mn, Mg) [27,28,29], and especially blended products, which are often of low quality and contaminated with heavy metals, this group of food products should be considered a significant source of toxic heavy metals for a general occupationally non-exposed population [30,31]. Because the consumption of black tea infusions in the studied population was high, it was hypothesized that the contribution of the group “Water and beverages” to the overall intake of some toxic metals would also be high.

A recent study performed in Brazil also indicated high consumption of cereals and meat (>3 servings per day) in the adult population. However, fruit proved to be the major group of food products, the consumption of which exceeded four servings per day, which seems normal, considering the climate and dietary habits among Brazilians [32]. A study performed among Australian adults also indicated that dairy products, followed by fruit, vegetables, and cereals, were consumed in the highest amounts. However, contrary to the results of the present research, fruit was consumed on a much higher level (a mean intake of 368.9 g/day) and meat in much lower amounts (89.6 g/day) [33]. This shows some serious dietary mistakes among the studied population and indicates the need for nutritional education in order to improve eating habits related to an increase in the consumption of vegetables and fruit, and a reduction of meat and meat products consumption, which seems crucial in the prevention of chronic non-communicable diseases, including cardio-vascular diseases, cancer, or diabetes [34,35,36].

### 3.1. Dietary Intake of Cd, Pb, Hg and Ni with Major Groups of Food Products

Until recently, the toxicological problems resulting from the presence of these metals in the environment concerned only a small group of people employed in specialized industries (the so-called occupational exposure). Currently, as a result of civilization changes, toxic heavy metals appear in high concentrations far beyond the emission sources. Their growing content in the environment causes disturbances in the biological balance of ecosystems, and their presence in the trophic chain creates conditions for the exposure of large groups of the population (the so-called environmental exposure) [37]. Currently, for a professionally unaffected person, food is the main source of exposure to toxic heavy metals such as Cd, Pb, Hg, or Ni [19,38]. As human activity causes their increased emission, anthropogenic factors contribute mainly to the increase in the content of these metals in nature, and food is a direct marker of changes in the content of these substances in the environment and human exposure.

The obtained results present the actual exposure to toxic heavy metals with food in the Polish adult population, including total daily intake and contribution of major food items the participation of which in the overall intake is the most significant. These data are presented in Table 3.

#### 3.1.1. Contribution of Major Food Items to an Overall Dietary Intake of Cd

Since the European Food Safety Authority (EFSA) in 2009 established a new tolerable weekly intake (TWI) value for Cd at 2.5 µg/kg b.w./week, which is significantly lower than the provisional tolerable weekly intake (PTWI) established by FAO/WHO in 1993 (7 µg/kg b.w./week) [8,39], it became clear that the daily intake, even in agricultural and uncontaminated areas, may be close to the limit values. Considering an average body weight of 58 and 74 kg for women and men of the investigated population, respectively, the daily intake of Cd with food should not exceed 20.7 and 26.4 µg, respectively. The obtained results reveal that the total intake of Cd was only 51.2% of the calculated PTWI in the case of women, and 49.1% in the case of men. These data are much lower in comparison to the intake of Cd with daily food rations determined previously in Poland—10.9–47.1 µg/day in 2006–2010 [2]; 12.7–21.6 µg/day in 2011–2013 [8], and 16.4–34.5 µg/day determined by Marzec and Schlegel-Zawadzka in diet duplicates in 1990–2002 [18]. The trend is decreasing, and it may suggest that the level of Cd in the environment is slowly declining. Present data also show that the potential risk of developing chronic toxicity through dietary exposure to Cd, even after a significant reduction of the PTWI value by EFSA, should be considered low. The performed study reveals that the actual dietary exposure to Cd in the Polish population is much lower compared to data for Asian countries–Lebanon (15.82 µg/day) [43], China (19.8 µg/day) [44], Japan (26.4 μg/day) [45], Vietnam (29–33 μg/day) [46], or Thailand (21–56 μg/day) [47], and it is rather close to the intake observed recently in European countries—Denmark (14–16 µg/day) [48,49], France (11.2 µg/day) [6], Sweden (10.6 µg/day) [50], or Serbia (11.51 µg/day) [51]. It should also be remembered that for some highly-industrialized areas, especially located in Asia, the observed daily Cd dietary intake was even 109–224 µg/day [52]. Moreover, scientific reports revealed that in some cases data from food analyses are lower than the Cd exposure estimated based on the urinary Cd excretion level [52]. However, a direct chemical analysis is considered a much better tool for the estimation of dietary exposure to Cd in comparison to calculations or data derived from databases (if these are available for particular regions or countries) [6,8,19]. Cd levels in foodstuffs and their overall dietary intake should be strictly controlled and monitored, considering its high toxicity and exceptionally long biological half-life [44]. Therefore, EC established strict regulations regarding acceptable limits for the content of Cd in various food products [4,5]. Moreover, even if the exposure is lower than recommended by EFSA or FAO/WHO guidelines, distinct pathologies in many organ systems are observed, which supports efforts to reduce the permissible limits of cadmium intake and continuous monitoring of the content of this element in food [52].

Cereals accounted for the largest share of the overall Cd intake, accounting for 30.2% and 39.6% of the total daily intake of Cd among women and men, respectively. Vegetables were the second major food group significantly contributing to Cd intake (17.4% and 21% among women and men, respectively), followed by sweets (11% and 9.25%), meat and meat products (10% and 9%), and potatoes (9.25% and 10.2%). The obtained results are fully in agreement with other data, pointing to cereals as the major contributors to the total dietary intake of Cd. According to the report prepared by EFSA, assessing in detail the Cd exposure from foodstuffs among adult Europeans, cereals were by far the main products, significantly contributing to the total intake of Cd (26.9%), followed by vegetables (16%), starchy roots and tubers (13.2%), and potatoes (13.2%). Some significant differences were also revealed between particular countries—potato consumption contributed close to a third of exposure in Irish adults (30%) and remained fairly high in many surveys except in Italy, Spain, and France [53]. The dietary intake of Cd with meat was especially high in Hungary (25%) and the Czech Republic (15%), while the contribution of fish was the highest among Spanish (31%) and Italian adults (23%) where consumption of these products is especially popular [53]. Cereals were found to be the major contributor to an overall Cd intake also in other European countries—Denmark (49%) [48,49], France (35%) [6], Spain (37.9%) [54], and Serbia (53.8%) [51]. Some of these studies also indicated vegetables and potatoes as the major contributors, after cereals [6,48,49,51]. Outside Europe, cereals are also a dominant contributor to Cd intake in Chile (37.9%) [55] or Asian countries, where Cd is mostly consumed with rice and vegetables, which is understandable if we consider the structure of food consumption in this region [44,45,46,47]. It seems that the structure of Cd consumption among Polish adults did not change significantly during the past 20 years, as Marzec and Schlegel-Zawadzka also indicated cereals, vegetables, and meat as the major sources of Cd in household diets for both genders [18]. However, some important differences may be observed—the contribution of meat and meat products is now significantly lower, and high amounts of Cd are consumed with sweets and sugars. While the reduced contribution of meat can be understood, which has been associated with the decline in consumption of this food in recent years (although in Poland it should be still considered high), the significant share of sugar and sweets may seem difficult to explain. It is hard to prove such results in the worldwide literature; however, Škrbić et al. [51] also indicated sugar as a significant contributor to the dietary intake of Cd in the Serbian population (16.3%).

#### 3.1.2. Contribution of Major Food Items to an Overall Dietary Intake of Pb

Similarly to Cd, the European Commission has established acceptable limits for the content of Pb in different food products [4,5]. Moreover, as in the case with Cd, in 2010, EFSA questioned the current value of PTWI (25 g/kg b.w.) as not providing complete safety for the population, as there was no evidence for a threshold for critical Pb-induced effects [8,42]. However, the new limit was not established. Instead, the CONTAM Panel identified a range of values for the 95% lower confidence limit of the benchmark dose of 1% extra risk (BMDL_01_) for each endpoint. The so-called BMDL values which establish the relationship between serum metal levels/dietary intake/the risk of intoxication may be briefly described as follows: a dietary intake of 0.50 µg/kg b.w.—a possibility of developing neurotoxicity in children; in the adult population—1.50 µg/kg b.w.—a potential risk of systolic blood pressure, and 0.63 µg/kg b.w.—a potential risk of developing chronic kidney disease. Evaluating the risk of intoxication related to the level of dietary exposure to Pb in the studied population, it was revealed that the dietary intake of Pb with daily food rations was 1.12 µg/kg b.w. among women and 1.02 µg/kg b.w. among men. These values suggest that the potential risk of developing nephrotoxicity should be considered high for both genders, and the potential risk due to its negative effect on blood pressure should be considered low. However, the lack of adequate data on nephrotoxicity among the studied population makes it difficult to draw broader conclusions about the impact of Pb intake with food on the development of chronic kidney disease. The obtained values are much higher compared to previously calculated exposure to Pb among adults in Poland (0.86 and 0.72 µg/kg among women and men, respectively) [8]. This indicates that despite the seemingly constant content of this metal in the environment, the nutritional risk related to Pb is still high. Over the past two decades, despite the reduction of Pb emissions with leaded petrol, the level of this metal in food products has remained at a similar level. It seems that this may be related to the increased combustion of solid fossil fuels in Asian countries (mainly in China and India) in recent years, which release very large amounts of Pb into the atmosphere and, as a result, this metal slowly enters Europe. Therefore, Pb toxicity is still a matter of great concern, as this metal is a well-known neurotoxicant, especially for children [56,57].

Studies regarding the dietary intake of Pb conducted in various European countries revealed that even among people occupationally non-exposed to Pb the risk of chronic intoxication is high. Coelho et al. assessed the exposure of a Portuguese academic community (the University of Aveiro, Portugal) to this metal through food consumption by evaluating its levels in duplicate diet samples. Pb was detected in all the analyzed samples with values ranging between 0.009 and 0.10 mg/kg, which corresponded to estimated daily intakes between 0.22 and 3.5 μg kg/b.w./day. An average exposure was estimated at 38.3 μg/person/day. Risk estimations disclose that at least 3.3% and 26.7% of the participants might experience cardiovascular and nephrotoxic effects, respectively [7]. In the former studies conducted in Poland, the mean dietary exposure to Pb was 66.5–106 μg/day [18] and 25.4–87.3 μg/day [8]. These values are close to the results obtained in the present study and approximate those determined in Italy [58], India [59], or Germany [60]. In contrast, Aung et al. revealed a very low intake in Japan (6.74 μg/day) [61], whereas data from China or Spain indicate a very high Pb intake (100–500 μg/day) [62,63]. This confirms the observations made by Coelho et al. [7] that the dietary intake of Pb varies significantly between regions and countries and may fall within a very wide range (from units to hundreds of μg per day).

Water and beverages were shown to be major contributors to the overall Pb intake in the present study, accounting for 25.3% and 31% of the total daily intake of Pb among women and men, respectively; followed by vegetables (18% and 15.8%), and meat and meat products (12.6% and 14.7%). Moreover, milk and dairy products were shown to be significant contributors to the daily intake of Pb among women (12.7%), and cereals among men (13%). The obtained results are in agreement with the EFSA report which indicated beverages (alcoholic and non-alcoholic beverages, water, and juices) as the main contributors. This group of food accounted, on average, for 27.8% of the total Pb intake in the European population, followed by grains and grain-based products (16.3%), milk and dairy products (10.6%), and vegetables (8.4%) [64]. A previous study conducted by Marzec and Schlegel-Zawadzka in Poland [16] revealed that cereals, vegetables, and meat were the main contributors to the total intake of Pb. However, the contribution of water and beverages (separately) was not evaluated. Therefore, to the best of our knowledge, the present study is the first to indicate (based on the analytical results) that water and beverages are the main contributors to the total Pb intake in the Polish adult population, which is fully in agreement with the present European trend.

Results of the present research show that Pb concentrations in the main food categories on the Polish market are at similar levels as in European data. However, since food is the major source of human exposure to Pb and there is no recommended tolerable intake level, the Pb content in food products should be kept as low as possible [7,64].

#### 3.1.3. Contribution of Major Food Items to an Overall Dietary Intake of Hg

According to EFSA, methylmercury (MeHg) is highly toxic, especially to the nervous system, and the developing brain is thought to be the most sensitive target organ for MeHg toxicity. While fish and seafood are the dominant sources of MeHg, other dietary products also contain Hg, but in a much lower toxic inorganic form. Therefore, while fish and seafood should be considered the only significant sources of MeHg to the body, it should not be forgotten that other products are also sources of exposure to Hg, although this form of the element does not pose such a high health risk [40]. Therefore, EFSA agreed that the PTWI established by JECFA (the Joint Food and Agriculture Organization of the United Nations and World Health Organization FAO/WHO Expert Committee on Food Additives) for total Hg (THg) as 4 µg/kg/b.w./week and only 1.6 µg/kg/b.w./week for MeHg are appropriate limits, even though the U.S. National Research Council (NRC) established an intake limit for MeHg of 0.7 μg/kg b.w./week, the 2.3-fold lower limit than that of JECFA [40,65,66,67].

The dietary intake of total mercury (THg) in the past 20 years in Poland has been very low, ranging from 1.90 to 7.07 µg/person/day [8,18]. In the latest paper on the dietary intake of Hg in the Polish population, Kuras et al. [68] revealed an average daily intake of 10.5 µg, but it was only based on fish consumption. No other products were included in the study, and, therefore, the intake of THg based on that research should be considered as significantly underestimated. The results of the present study are much higher compared to previous Polish data and similar to other European results where the THg intake was estimated in a very wide range of 1.38–40.2 µg/person/day [6,8,19]. Assuming that all Hg contained in fish products is MeHg, the dietary intake of MeHg contributes only to 14.6 and 15.8% of the appropriate PTWI value for MeHg. These values are much lower in comparison to previous Polish results [68]. However, it should be remembered that fish consumption revealed by Kuras et al. [68] was 2–3 times higher in comparison to the present results and, therefore, the contribution of fish to the overall Hg intake in the present research was very low. The MeHg intake is strictly correlated with the amount of fish consumed, and this varies significantly between studies and subpopulations, as previous Polish studies revealed the level of only 3.2–7.89% of the PTWI for MeHg, including sea fish and fish from Polish lakes [69,70,71]. According to EFSA, the level of MeHg intake depends not only on the amount of fish consumed but also on the concentration of this element in fish tissue. Previous studies conducted in Poland revealed that the average Hg concentration in fish from the Polish market was 35–54 µg/kg and it was far below the limits established in the European Union (EU) for this toxic metal (0.5 mg/kg for Hg) [4,5,68,69,70]. In the present study, the average concentration of Hg in fish products was much higher (122 µg/kg), but still far below the EU limits.

The dietary intake of THg in the present research contributes only to 59.8 and 51.8% of the PTWI for THg for women and men, respectively. Moreover, considering that, according to the EFSA statement, only MeHg poses a significant dietary risk, the Hg intake from sources other than food and sea products is much less important [66]. In the present study, cereals (15.2 and 16.8% among women and men, respectively), vegetables (19.6 and 16.1%), and milk products (18.2 and 15.4%) were the main contributors to the overall Hg intake. This is in agreement with previous studies by Marzec and Schlegel-Zawadzka [18], who also indicated these groups of foods as contributing mostly to the general Hg intake. Interestingly, the former Polish study also revealed that although fish contain high concentrations of Hg, because of the very low intake among Poles, the participation of these products in the THg intake is insignificant. The present study shows that this trend has not changed during the past 20 years, and fish consumption in the Polish population remains very low. However, as mentioned before, this parameter is very variable and differs significantly between the subpopulations included in the study.

#### 3.1.4. Contribution of Major Food Items to an Overall Dietary Intake of Ni

Excluding some particularly sensitive individuals, the risk of developing toxicity through dietary intake of Ni among the general population should be considered low [72]. The previous TDI (tolerable daily intake) value of 300 µg/person established by WHO in 1993 [73] was recently changed by EFSA and increased to 13 µg/kg b.w. [41]. The mean dietary intake of Ni in the present study was 3.89 µg/kg b.w./day in the group of women and 2.93 µg/kg b.w./day among men, which was far below an updated TDI value. These values are higher than those previously reported in Poland—1.68–2.42 µg/kg b.w./per day for women and 1.99–2.60 µg/kg b.w./day for men [2]. In another study conducted on Polish adults, the mean dietary exposure to Ni among women was 384 µg/day and among men 455 µg/day. The corresponding exposures related to body weight were 6.18–6.82 µg/kg b.w./day (women; range) and 6.30–8.09 µg/kg b.w./day (men; range) [6,39]. Bartos et al. [74] observed a mean Ni intake of 227 µg/day in a group of Polish students aged 20–25 years, a level close to the results of the present study. Studies on the Ni intake with daily food conducted recently in other European countries confirm the latest statement of EFSA that there is no risk of Ni toxicity via food consumption, by either chronic or acute exposure [41]. Suomi et al. [75] evaluated a dietary exposure to Ni among Finnish adults at 2.53 µg/kg b.w./day. In the Italian Total Diet Study (TDS), the mean dietary intake of Ni was from 1.5 to 4.6 µg/kg b.w./day across age classes [76]. In the 2014 TDS carried out in the UK, Ni did not pose a significant nutritional risk for adults, and the highest exposure was estimated for children aged 1.5–3 years [77].

Cereals were shown to be by far the major contributor to the overall Ni intake in the present study, accounting for 38.1% and 40.4% of the total daily intake of Ni, among women and men, respectively; followed by vegetables (19.3% and 13.5%), water and beverages (11.7% and 18.9%), and sweets and sugars (8.22% and 11.8%). The obtained results are similar to other reports on the structure of Ni intake with various foods. EFSA indicated cereals as the major contributor to the total Ni intake, followed by non-alcoholic beverages and vegetables. For adult age groups, coffee beverages were the main contributor, and soft drinks and cocoa beverages for toddlers, other children, and adolescents [41]. A Finnish study revealed that ‘Cereals and cereal products’, ‘Legumes, nuts and seeds’, and ‘Sugar and sweets’ were the main contributors to the total Ni intake among people aged 25–64 years [75]. A recent Italian study provided similar results—the main contributors to the dietary exposure to Ni were cereals and cereal products (27%), sweet products (16%), vegetables (11%), potatoes (8%), fruit (7%), and pulses (6%) [41,76]. A study by Larsen et al. [72] showed that beverages, followed by cereals and milk products, were the dominant contributors to the overall Ni intake. According to a recent EFSA report [41], the contribution of particular food groups to the overall Ni intake varied between the dietary surveys, which may be explained by the specific food consumption patterns in individual European countries and even in different regions within a country. In general, a high contribution of cereals is linked to high consumption observed in many countries and not to especially high contamination with Ni. In addition, despite relatively high Ni concentrations measured in ‘Herbs, spices and condiments’ and ‘Products for special nutritional use’, the exposure to these foods was small considering the very low consumption recorded within the dietary surveys [41]. This is in agreement with the results of the present study where the highest contribution to the Ni exposure was revealed for products consumed in the highest amounts.

The present results confirm that the Ni intake has remained at a similar level in various countries during the past 20 years and the structure of food groups that contribute the most to the Ni intake has not changed significantly. It was also revealed that there is no health risk regarding exposure to Ni from food products.

#### 3.1.5. Principal Component Analysis (PCA)

Based on the results obtained, a matrix made of columns (elements) and rows (products) was created and subjected to PCA.

**Figure 1:**
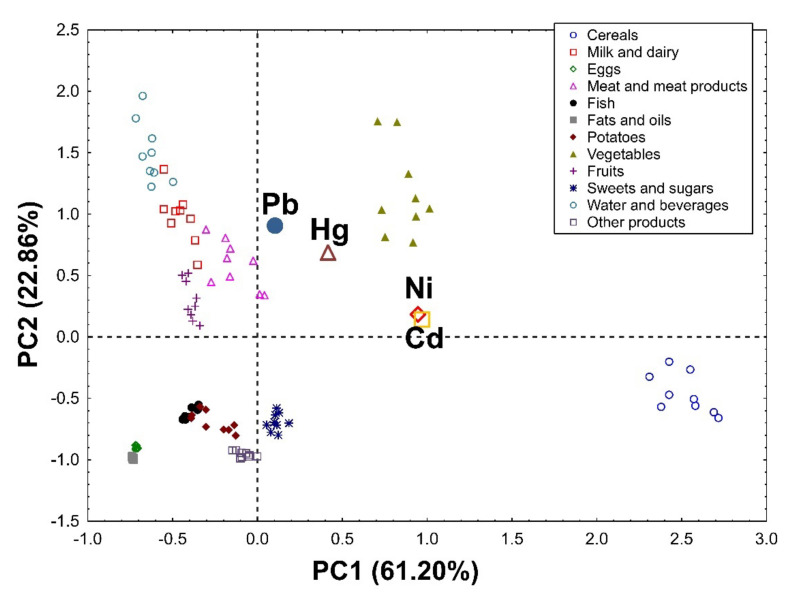
PCA biplot showing loadings (for heavy metals) and scores (for products) of the first two principal components of PCA in the group of women, explaining together 84.06% of the variability in the obtained dataset (61.20% and 22.86% in PC1 and PC2, respectively).

**Figure 2:**
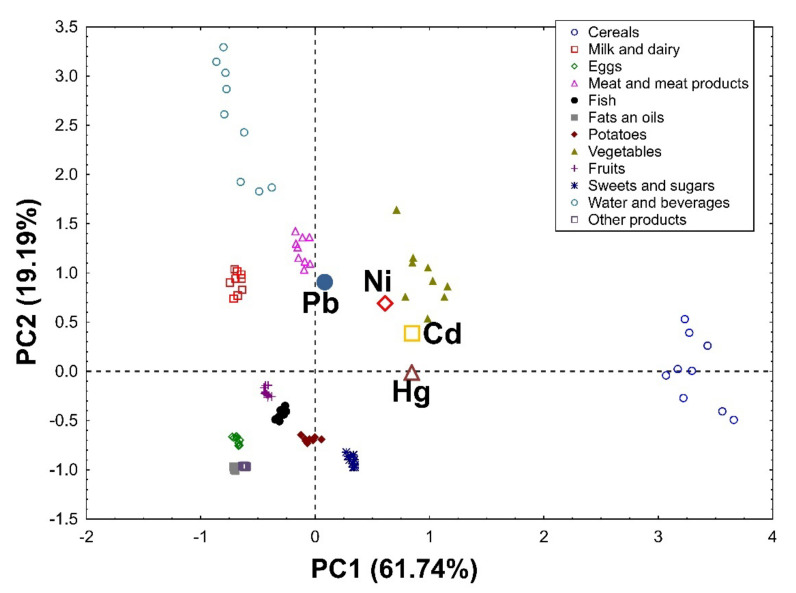
PCA biplot showing loadings (for heavy metals) and scores (for products) of the first two principal components of PCA in the group of men, explaining together 80.93% of the variability in the obtained dataset (61.74% and 19.19% in PC1 and PC2, respectively).

Applying Principal Component Analysis to the data set, it was possible to extract two significant, cross-validated principal components that accounted for 84.06% and 80.93% of the variability in the original data in the female (Figure 1) and male groups (Figure 2), respectively.

In the group of women, PC1 was associated mainly with the total intake of heavy metals and divided the investigated products into the first group (cereals and vegetables) as products contributing the most to the overall heavy metal intake, and the second group (eggs, fats, and oils) as products participating very little in the general intake of these elements. PC2 distinguishes water and beverages, vegetables, milk, and dairy products as those contributing the most to the dietary intake of Pb and Hg (Figure 1).

In the group of men, PC1 is also associated with the total supply of heavy metals and, first of all, it distinguishes the first group (cereals and vegetables) as products supplying the largest amounts of heavy metals, and the second group (eggs, fats and oils, and other products) as products providing the smallest amounts of these elements. In addition, it was revealed that cereals contributed the most to the daily intake of Hg and Cd. PC2 definitely distinguishes water and beverages as products that provide the highest amounts of Pb and a lot of Ni. Moreover, large amounts of Pb were delivered with vegetables, meat, and meat products, as well as milk and dairy products (Figure 2). In addition, vegetables, meat, and meat products significantly contributed to the total intake of Ni. It was also confirmed that, despite containing high amounts of Hg, considering their very low consumption, fish were not an important source of this element for the general population in the present study.

## 4. Conclusions

This research evaluated the risk of intoxication with various heavy metals from different foodstuffs, using the 24-dietary recall technique with analytical determinations (ICP-OES). The obtained results reveal that there is a high potential risk of developing nephrotoxicity through the dietary intake of Pb, in the case of both genders. However, appropriate data on biochemical parameters and clinical symptoms of nephrotoxicity are needed to fully confirm the impact of Pb intake on the development of chronic kidney disease. The dietary intake determined for other elements (Cd, Hg, and Ni) was far below the limits established by EFSA. PCA supported the analytical determinations and revealed that cereals and vegetables were the major contributors to the total intake of Cd, Ni, and Hg, and water and beverages were the major dietary sources of Pb. Based on the PCA results it was also shown that meat and meat products contributed significantly to the overall dietary intake of Ni. In contrast, eggs, fats, and oils, as well as other products provided the smallest amounts of Cd, Pb, and Ni.

## Figures and Tables

**Table 1:** Composition of food groups among women and men * [20].

Food Group	Women			Men		
	Amount (g)	Energy (kcal)	Composition (%)	Amount (g)	Energy (kcal)	Composition (%)
Cereals	224.1 ± 20.8	653.6 ± 79.2	baking 51.5 cereal flakes 14.7 grits 12.5 rice 11.5 pasta 9.80	284.8 ± 25.3	770.1 ± 84.2	baking 66.6 rice 12.9 pasta 9.40 cereal flakes 6.35 grits 4.75
Milk and dairy	276.9 ± 25.7	303.9 ± 35.4	milk 45.1 cheese 30.4 yogurts 23.2 sour cream 1.3	232.3 ± 22.4	327.1 ± 29.8	cheese 47.0 milk 31.2 yogurts 18.9 sour cream 2.90
Eggs	18.2 ± 2.11	21.4 ± 1.92	scrambled eggs 48.1 boiled eggs 38.7 omlette 13.2	45.5 ± 5.17	53.1 ± 7.12	scrambled eggs 60.9 boiled eggs 33.2 omlette 5.90
Meat and meat products	191.7 ± 20.8	406.5 ± 47.2	pork 39.0 poultry 37.6 beef 23.4	283.5 ± 31.4	621.5 ± 56.4	pork 42.0 beef 30.4 poultry 27.6
Fish	17.5 ± 2.14	21.4 ± 2.72	tuna 44.2 cod 38.6 salmon 12.0 herring 4.0 pike 1.2	20.0 ± 3.18	36.7 ± 4.12	mackerel 47.0 salmon 35.3 tuna 8.90 sardine 8.80
Fats and oils	14.0 ± 1.34	116.7 ± 11.4	butter 35.2 rapeseed oil 32.5 sunflower oil 15.7 olive oil 11.1 margarine 3.29 linseed oil 2.21	25.1 ± 2.91	209.7 ± 18.4	rapeseed oil 38.2 butter 32.6 sunflower oil 20.2 lard 6.90 olive oil 2.10
Potatoes	50.1 ± 6.55	38.6 ± 4.22		82.5 ± 9.52	63.5 ± 7.48	
Vegetables	384.1 ± 45.2	90.9 ± 12.5	tomatoes 30.1 pepper 19.4 carrot 10.9 cabbage 9.95 beetroot 7.85 cauliflower 6.82 broccoli 6.64 lettuce 2.17 celery 1.16 others 5	343.8 ± 38.4	72.6 ± 9.15	tomatoes 32.2 carrot 22.3 pepper 15.6 cucumber 11.2 onion 4.05 cabbage 3.58 broccoli 2.50 pumpkin 1.30 leek 1.27 lettuce 1.05 others 5
Fruits	227.2 ± 35.6	140.5 ± 18.8	apples 34.2 bananas 33.6 oranges 7.65 mandarins 5.92 pears 4.14 peaches 3.42 raspberries 3.05 watermelons 1.86 kiwi 1.48 raisins 1.32 nectarines 1.25 others 1.44	192.2 ± 26.2	114.4 ± 17.8	apples 40.5 bananas 28.9 oranges 10.1 mandarins 5.92 pears 5.56 grapefruit 3.10 grapes 1.55 red currants 1.85 dried dates 1.36 others 1.16
Sweets and sugars	62.4 ± 10.2	236.9 ± 31.8	cakes and cookies 53.4 bars 14.3 sugar 13.4 chocolate 12.5 honey 1.70 others 4.70	69.1 ± 12.4	251 ± 40.8	cakes and cookies 60.5 sugar 23.4 bars 10.6 chocolate 4.80 honey 0.70
Water and beverages	638.7 ± 75.4	33.8 ± 4.52	black tea (infusion) 56.8 soups (only water) 18.8 fruit juices 11.1 coffee (infusion) 11.0 sweet beverages 2.30	713.1 ± 100.2	52.3 ± 6.18	black tea (infusion) 53.4 coffee (infusion) 17.0 soups (only water) 13.2 fruit juices 11.8 sweet beverages 3.27 bear 1.33
Other products	34.5 ± 5.52	139 ± 15.8	jams (no added sugar) 32.4 sauces 22.8 hooves 16.9 seeds (pumpkin/sunflower) 10.6 nuts 5.50 snacks 4.24 pudding 2.56 almonds 2.11 tomato concentrate 1.94 cocoa 0.95	18.4 ± 3.11	98.7 ± 10.2	sauces 56.3 jams (no added sugar) 22.0 pancakes 13.4 almonds 2.78 nuts 2.25 pudding 1.68 cocoa 1.10 others 0.49
Total	2139 ± 264	2203 ± 331		2310 ± 351	2671 ± 355	

* average data based on the 3-days dietary recall.

**Table 2:** Selective validation parameters of the applied method.

Parameter	Cd	Pb	Hg	Ni
Reference value (mg/kg)	0.30 ± 0.02	0.50 ± 0.04	0.20 ± 0.01	0.50 ± 0.03
Determined value (mg/kg)	0.27	0.53	0.16	0.52
	0.31	0.48	0.18	0.51
	0.33	0.46	0.16	0.57
	0.29	0.52	0.21	0.49
	0.27	0.52	0.23	0.55
	0.28	0.54	0.17	0.5
Average	0.29	0.51	0.19	0.54
SD	0.02	0.03	0.03	0.03
RSD (%)	8.28	6.08	13.68	5.58
Recovery (%)	96.67	102.0	95.0	106.0
LOD * (µg/kg)	0.8	4.5	1.8	1.2
LOQ ** (µg/kg)	2.1	14.8	6.0	4.0

* LOD—Limit of Detection; ** LOQ—Limit of Quantification.

**Table 3:** Daily intake of toxic heavy metals with particular food groups. Each value represents mean ± SD, min, and max values. The means not sharing the same letter in a column are significantly different at *p* ≤ 0.05. The underlined means are significantly different between women and men at *p* ≤ 0.05.

Food Group	Element							
	Cd (µg)		Pb (µg)		Hg (µg)		Ni (µg)	
	Women	Men	Women	Men	Women	Men	Women	Men
Cereals	**3.20 ± 0.25 ^g^** 2.82–3.46	**5.13 ± 0.25 ^f^** 4.71–5.44	**5.28 ± 0.94 ^c^** 4.12–6.76	**9.80 ± 1.97 ^cd^** 7.26–12.91	**2.99 ± 0.23 ^g^** 2.71–3.42	**3.57 ± 0.24 ^i^** 3.21–3.84	**85.73 ± 6.34 ^g^** 77.89–95.25	**87.57 ± 6.78 ^g^** 74.21–94.87
Milk and dairy	**0.45 ± 0.07 ^bc^** 0.36–0.55	<LOQ	**8.26 ± 1.18 ^d^** 6.33–10.19	**7.72 ± 0.48 ^d^** 7.05–8.34	**3.59 ± 0.26 ^h^** 3.11–3.91	**3.27 ± 0.26 ^h^** 2.81–3.55	**3.37 ± 0.35 ^ab^** 2.75–3.85	**3.07 ± 0.41 ^ab^** 2.55–3.58
Eggs	**0.06 ± 0.01 ^a^** 0.05–0.07	**0.12 ± 0.01 ^a^** 0.10–0.13	**0.87 ± 0.07 ^a^** 0.76–0.95	**1.72 ± 0.31 ^ab^** 1.31–2.21	**0.22 ± 0.02 ^a^** 0.19–0.25	**0.51 ± 0.09 ^cd^** 0.34–0.62	**0.50 ± 0.06 ^a^** 0.40–0.59	**1.30 ± 0.10 ^a^** 1.18–1.50
Meat and meat products	**1.06 ± 0.17 ^e^** 0.77–1.28	**1.17 ± 0.09 ^d^** 1.03–1.33	**8.16 ± 1.10 ^d^** 6.55–9.45	**11.03 ± 0.78 ^c^** 9.84–12.07	**2.59 ± 0.25 ^f^** 2.21–2.94	**3.48 ± 0.27 ^hi^** 3.05–3.92	**10.0 ± 0.96 ^c^** 8.55–11.62	**10.52 ± 0.98 ^d^** 9.11–12.11
Fish	**0.29 ± 0.02 ^b^** 0.26–0.34	**0.38 ± 0.03 ^bc^** 0.34–0.43	**0.40 ± 0.06 ^a^** 0.32–0.48	**0.65 ± 0.08 ^a^** 0.53–0.76	**1.94 ± 0.22 ^d^** 1.71–2.34	**2.67 ± 0.21 ^g^** 2.40–3.0	**0.67 ± 0.08 ^a^** 0.56–0.80	**0.38 ± 0.04 ^a^** 0.31–0.43
Fats and oils	<LOQ	<LOQ	**0.29 ± 0.04 ^a^** 0.22–0.35	**0.97 ± 0.10 ^a^** 0.81–1.12	**0.14 ± 0.02 ^a^** 0.11–0.17	**0.15 ± 0.02 ^a^** 0.12–0.18	**0.16 ± 0.01 ^a^** 0.14–0.17	**1.58 ± 0.24 ^ab^** 1.22–1.94
Potatoes	**0.98 ± 0.21 ^e^** 0.69–1.26	**1.32 ± 0.13 ^d^** 1.18–1.54	**2.98 ± 0.35 ^b^** 2.56–3.55	**2.99 ± 0.24 ^b^** 2.70–3.28	**0.50 ± 0.05 ^bc^** 0.42–0.57	**0.82 ± 0.06 ^e^** 0.72–0.87	**4.88 ± 0.35 ^b^** 4.35–5.27	**5.89 ± 0.49 ^bc^** 5.18–6.59
Vegetables	**1.84 ± 0.26 ^f^** 1.53–2.11	**2.73 ± 0.26 ^e^** 2.41–3.09	**11.65 ± 2.06 ^e^** 9.32–15.0	**11.88 ± 1.96 ^c^** 8.51–15.56	**3.87 ± 0.26 ^i^** 3.52–4.20	**3.42 ± 0.26 ^hi^** 3.03–3.85	**43.43 ± 3.18 ^f^** 40.18–49.58	**29.18 ± 2.19 ^e^** 26.14–32.18
Fruits	**0.26 ± 0.03 ^ab^** 0.22–0.30	**0.22 ± 0.03 ^ab^** 0.18–0.29	**5.77 ± 0.90 ^c^** 4.50–7.04	**3.12 ± 0.20 ^b^** 2.74–3.32	**2.32 ± 0.15 ^e^** 2.08–2.55	**1.89 ± 0.12 ^f^** 1.70–2.10	**12.42 ± 0.52 ^c^** 11.45–12.92	**7.77 ± 0.76 ^cd^** 6.75–9.14
Sweets and sugars	**1.17 ± 0.08 ^e^** 1.04–1.28	**1.20 ± 0.09 ^d^** 1.10–1.35	**3.13 ± 0.48 ^b^** 2.32–3.84	**1.98 ± 0.24 ^ab^** 1.62–2.35	**0.75 ± 0.07 ^b^** 0.65–0.85	**0.76 ± 0.09 ^de^** 0.64–0.86	**18.48 ± 1.15 ^d^** 16.32–19.61	**25.63 ± 1.84 ^e^** 23.12–28.16
Water and beverages	**0.59 ± 0.09 ^cd^** 0.44–0.69	**0.54 ± 0.07 ^c^** 0.45–0.67	**16.37 ± 1.59 ^f^** 14.33–19.36	**23.25 ± 3.52 ^e^** 18.87–27.99	**0.54 ± 0.04 ^b^** 0.49–0.63	**0.45 ± 0.07 ^bc^** 0.36–0.58	**26.27 ± 2.35 ^e^** 22.14–29.15	**41.09 ± 5.90 ^f^** 30.17–47.87
Other products	**0.70 ± 0.07 ^d^** 0.62–0.84	**0.15 ± 0.02 ^a^** 0.12–0.18	**1.59 ± 0.11 ^ab^** 1.42–1.74	**0.63 ± 0.03 ^a^** 0.58–0.65	**0.27 ± 0.03 ^ac^** 0.23–0.32	**0.21 ± 0.02 ^ab^** 0.18–0.23	**18.85 ± 1.61 ^d^** 16.11–20.78	**2.88 ± 0.34 ^ab^** 2.32–3.24
Total	10.59 ± 0.74 9.74–11.67	12.97 ± 0.36 12.40–13.49	64.82 ± 2.79 61.29–69.57	75.12 ± 4.58 69.31–82.11	19.73 ± 0.67 18.43–20.54	21.19 ± 0.75 20.37–22.33	224.76 ± 9.64 210.23–236.68	216.86 ± 8.20 205.82–227.41
PTWI/TWI/TDI (µg) [8,39,40,41,42]	20.7	26.4	NA *	NA *	33 (THg)	42.3 (THg)	754	962

* NA (not applicable)—lack of a current PTWI value for lead, means and SD values were presented in bold.

## Data Availability

The data presented in this study are available in the present article.
